# Selenium and Selenoprotein Deficiencies Induce Widespread Pyogranuloma Formation in Mice, while High Levels of Dietary Selenium Decrease Liver Tumor Size Driven by TGFα

**DOI:** 10.1371/journal.pone.0057389

**Published:** 2013-02-27

**Authors:** Mohamed E. Moustafa, Bradley A. Carlson, Miriam R. Anver, Gerd Bobe, Nianxin Zhong, Jerrold M. Ward, Christine M. Perella, Victoria J. Hoffmann, Keith Rogers, Gerald F. Combs, Ulrich Schweizer, Glenn Merlino, Vadim N. Gladyshev, Dolph L. Hatfield

**Affiliations:** 1 Molecular Biology of Selenium Section, Laboratory of Cancer Prevention, National Cancer Institute, National Institutes of Health, Bethesda, Maryland, United States of America; 2 Pathology/Histotechnology Laboratory, Science Applications International Corporation-Frederick, Inc., Frederick National Laboratory for Cancer Research, Frederick, Maryland, United States of America; 3 Department of Animal and Rangeland Sciences, College of Agriculture, and Linus Pauling Institute, Oregon State University, Corvallis, Oregon, United States of America; 4 Immunopathology Section, National Institute of Allergy and Infectious Diseases, National Institutes of Health, Bethesda, Maryland, United States of America; 5 Laboratory Animal Sciences Program, Science Applications International Corporation-Frederick, Inc., Frederick National Laboratory for Cancer Research, Frederick, Maryland, United States of America; 6 Office of the Director, Diagnostic and Research Services Branch, Center for Cancer Research, National Cancer Institute, National Institutes of Health, Bethesda, Maryland, United States of America; 7 Grand Forks Human Nutrition Research Center, Grand Forks, North Dakota, United States of America; 8 Charite-Universitatsmedizin Berlin, Institut fur Experimentelle Endokrinologie, Berlin, Germany; 9 Laboratory of Cancer Biology and Genetics, National Cancer Institute, National Institutes of Health, Bethesda, Maryland, United States of America; 10 Division of Genetics, Department of Medicine, Brigham & Women’s Hospital and Harvard Medical School, Boston, Massachusetts, United States of America; Klinikum rechts der Isar der TU München, Germany

## Abstract

Changes in dietary selenium and selenoprotein status may influence both anti- and pro-cancer pathways, making the outcome of interventions different from one study to another. To characterize such outcomes in a defined setting, we undertook a controlled hepatocarcinogenesis study involving varying levels of dietary selenium and altered selenoprotein status using mice carrying a mutant (A37G) selenocysteine tRNA transgene (*Trsp^tG37^*) and/or a cancer driver *TGFα* transgene. The use of *Trsp^tG37^* altered selenoprotein expression in a selenoprotein and tissue specific manner and, at sufficient dietary selenium levels, separate the effect of diet and selenoprotein status. Mice were maintained on diets deficient in selenium (0.02 ppm selenium) or supplemented with 0.1, 0.4 or 2.25 ppm selenium or 30 ppm triphenylselenonium chloride (TPSC), a non-metabolized selenium compound. *Trsp^tG37^* transgenic and *TGFα*/*Trsp^tG37^* bi-transgenic mice subjected to selenium-deficient or TPSC diets developed a neurological phenotype associated with early morbidity and mortality prior to hepatocarcinoma development. Pathology analyses revealed widespread disseminated pyogranulomatous inflammation. Pyogranulomas occurred in liver, lungs, heart, spleen, small and large intestine, and mesenteric lymph nodes in these transgenic and bi-transgenic mice. The incidence of liver tumors was significantly increased in mice carrying the *TGFα* transgene, while dietary selenium and selenoprotein status did not affect tumor number and multiplicity. However, adenoma and carcinoma size and area were smaller in *TGFα* transgenic mice that were fed 0.4 and 2.25 versus 0.1 ppm of selenium. Thus, selenium and selenoprotein deficiencies led to widespread pyogranuloma formation, while high selenium levels inhibited the size of *TGFα*–induced liver tumors.

## Introduction

Selenium has been reported to have a role in cancer prevention as well as roles in preventing various maladies (e.g., heart disease and other cardiovascular and muscle disorders, inhibiting viral expression, delaying the onset of AIDS in HIV positive patients, slowing the aging process, and roles in mammalian development, male reproduction and immune function) [1]. Although there has been considerable debate in the selenium field as to whether small molecular weight selenocompounds or selenoproteins are responsible for these health benefits, studies suggest that both classes of these components play roles [2]. The available evidence, however, suggests that selenoproteins may have a more prominent role in providing health benefits (reviewed in [3]).

Mice are the most commonly used animal models to study the role of selenium in health and disease. These models are amenable to dietary interventions and various gene manipulations. Typically, diets varying in selenium (in the form of selenite or selenomethionine) are used, covering the range from deficiency to excess of this essential micronutrient. In addition, triphenylselenonium chloride (TPSC), which is known to mediate cancer [4], is a low molecular weight selenium-containing compound (selenocompound). TPSC is not metabolized to release selenium and it has also been used in diet studies involving mouse cancer models [4].

Several mouse models have been devised to examine the role of all selenoproteins, stress-related selenoproteins or individual selenoproteins in health and development as well as in cancer prevention (see reviews [5]–[7]). These mouse models include the targeted removal of the selenocysteine (Sec) tRNA^[Ser]Sec^ gene (designated *Trsp*) in specific tissues or organs, mutant Sec tRNA^[Ser]Sec^ transgenes and the targeted removal of specific selenoproteins that may involve subjecting the resulting mice to environmental stresses and/or specific cancer driver genes.

Expression of selenoproteins is dependent on the presence Sec tRNA^[Ser]Sec^ and the targeted removal of the gene encoding this tRNA, *Trsp*, is embryonic lethal (see [5] and references therein). There are two isoforms of Sec tRNA^[Ser]Sec^, methylcarboxymethyl-5′-uridine (mcm^5^U) and methylcarboxymethyl-5′-uridine-2′-*O*-methylribose (mcm^5^Um), that differ from each other by only the 2′-*O*-methyl group on the ribosyl moiety at position 34 (Um34) (reviewed in [5]). Interestingly, mcm^5^U is closely linked to the expression of housekeeping selenoproteins (e.g., thioredoxin reductase 1–3 [TR1-3]), while mcm^5^Um is closely linked with expression of stress-related selenoproteins (e.g., glutathione peroxidase 1 [GPx1] and selenoprotein W [SelW]). In addition, the expression of mcm^5^Um is sensitive to Se status. Mutations in Sec tRNA^[Ser]Sec^ that affect base modifications in the anticodon loop also prevent Um34 synthesis; thus, dramatically reducing stress-related selenoprotein expression [5]. Sec tRNA^[Ser]Sec^mutants have been used as mouse models to generate mice lacking stress-related selenoproteins that in turn have been used to investigate the role of selenoproteins in cancer prevention (see [5] and references therein).

Earlier studies involving selenium and tumor formation examined the role of dietary selenium on liver cancer in *TGFα/c-Myc* mice, wherein hepatocarcinogenesis was suppressed by both selenium deficiency and high levels of selenium [8]. Selenium deficiency has also been reported to play a role in preventing other cancers [9], whereas reduced stress-related selenoprotein expression and selenium deficiency have been shown to play a role in enhancing colon [10] and prostate cancers [11]. The total loss of selenoprotein expression in mouse mammary epithelial cells has also been found to enhance breast cancer [12]. Thus, selenium and selenoprotein deficiencies play roles in enhancing some cancers, while selenium and selenoproteins present in normal levels play roles in preventing other cancers.

Herein, we further explored the role of selenoproteins and dietary selenium in liver cancer by employing transgenic mice that carried a mutant *Trsp* transgene lacking an *N^6^*-isopentenyladenosine (i^6^A) at position 37 (A37G) of Sec tRNA^[Ser]Sec^ (designated *Trsp^tG37^*) [13]. As noted above, the A37G mutant Sec tRNA^[Ser]Sec^ does not synthesize Um34 [14], [15], and as a consequence, *Trsp^tG37^* animals are deficient in expression of stress-related selenoproteins independent of their dietary selenium status. We fed these mice Torula yeast-based selenium-deficient diets supplemented with 0, 0.1, 0.4 and 2.25 ppm selenium as sodium selenite or 30 ppm selenium as TPSC. We also employed mice that carried a liver cancer driver transgene, *TGFα* [16], and used bi-transgenic mice encoding both *TGFα*/*Trsp^tG37^* transgenes. Mice carrying *Trsp^tG37^* that were maintained on a selenium-deficient or TPSC supplemented diets were, independent of *TGFα*, extremely selenium-deficient and developed early morbidity and mortality. They manifested a neurological phenotype reminiscent of mouse models with severely impaired cerebral selenoprotein expression [17]–[21]. In addition, pathology analysis uncovered a high incidence of pyogranulomous inflammation associated with selenium deficiency. Pyogranulomas are a nodular inflammatory lesion with both a suppurative component and a epithelioid macrophage component with an outer rim of mononuclear leukocytes. Dietary selenium or selenoprotein status did not significantly affect tumor incidence and multiplicity but high levels of dietary selenium (0.4 or 2.25 versus 0.1 ppm) inhibited tumor size in *TGFα*-driven hepatocarcinogenesis.

## Methods

### Ethics Statement

All mouse experiments were approved by the Animal Ethics Committee at the National Institutes of Health and mice were handled and humanely sacrificed in strict accordance with the National Institutes of Health Institutional Guidelines (NCI, NIH, Bethesda, MD, USA). NCI-Frederick is accredited by AAALAC International and follows the Public Health Service Policy for the Care and Use of Laboratory Animals. Animal care was provided in accordance with the procedures outlined in the *Guide for Care and Use of Laboratory Animals* (National Research Council; 2011, National Academy Press, Washington, D.C.).

### Materials

Selenium-75 (^75^Se) (specific activity 1000 Ci/mmol) was purchased from the Research Reactor Facility, University of Missouri (Columbia, MO) and [^3^H]serine (specific activity 29 Ci/mmol) and Hybond Nylon N^+^ membranes from Amersham Biosciences. NuPage 10% polyacrylamide gels and polyvinylidene difluoride (PVDF) membranes were obtained from Stratagene and the Torula yeast based diets [22] were supplied by Harlan Teklad. All other reagents were obtained commercially and were products of the highest grade available.

### Mice, Genotyping of Mice and Diets

Male mice on a FVB/N background that encoded 20 copies of the *Trsp^tG37^* transgene were genotyped as reported [13]. *TGFα* mice on a CD1 background were obtained and genotyped as given [16]. Mice homozygous for *Trsp^tG37^* were mated with wild type CD1 mice to obtain offspring that were heterozygous for *Trsp^tG37^* (*Trsp^tG37^*/+). Mice homozygous for *TGFα* were mated with the wild type FVB strain to obtain offspring that were heterozygous for *TGFα* (*TGFα*/+). Mice homozygous for *TGFα* were mated with mice homozygous for *Trsp^tG37^* to obtain offspring that were heterozygous for both transgenes (*TGFα*/*Trsp^tG37^*). Male offspring at 4 weeks of age that were obtained from each of the above matings were placed on separate Torula yeast based diets as described below (see also Table 1). A control group consisting of wild type mice on the FVB/CD1 background was obtained by breeding wild type FVB/N and wild type CD1 mice (CD1 mice (from Charles Rivers, Wilmington, MA). A minimum of 6 mice from the control group breedings were placed on Torula yeast based diets (see below).

**Table 1:** Experimental design^a^.

Groups and Subgroups	Number of mice per Group	Genotype	Diet	Average days on the diet^b^
1A	20	*TGFα*/*Trsp^tG37^*	0.02 ppm Se	126
1B	14	*TGFα*/*Trsp^tG37^*	0.1 ppm Se	501
1C	27	*TGFα*/*Trsp^tG37^*	0.4 ppm Se	409
1D	19	*TGFα*/*Trsp^tG37^*	2.25 ppm Se	478
1E	28	*TGFα*/*Trsp^tG37^*	30 ppm Se as TPSC	134
2A	18	*Trsp^tG37^*/*+*	0.02 ppm Se	110
2B	13	*Trsp^tG37^*/*+*	0.1 ppm Se	444
2C	19	*Trsp^tG37^*/*+*	0.4 ppm Se	434
2D	19	*Trsp^tG37^*/*+*	2.25 ppm Se	363
2E	12	*Trsp^tG37^*/*+*	30 ppm Se as TPSC	236
3A	21	*TGFα*/*+*	0.02 ppm Se	392
3B	21	*TGFα*/*+*	0.1 ppm Se	453
3C	20	*TGFα*/*+*	0.4 ppm Se	525
3D	19	*TGFα*/*+*	2.25 ppm Se	514
3E	18	*TGFα*/*+*	30 ppm Se as TPSC	477
4A	10	*+*/*+*	0.02 ppm Se	416
4B	6	*+*/*+*	0.1 ppm Se	435
4C	8	*+*/*+*	0.4 ppm Se	333
4D	9	*+*/*+*	2.25 ppm Se	495
4E	9	*+*/*+*	30 ppm Se as TPSC	302

a The study covered a period of 18 months wherein mice in 1A, 1E, 2A and 2E died or were sacrificed at earlier time points as noted in the text and see Table 2.

b Time on the diet before death or euthanasia.

There were four major groups, Groups 1–4, consisting of five Subgroups, A–E, wherein each Subgroup was placed on different levels of dietary selenium or a single level of TPSC (Table 1). The levels of selenium in Subgroups A–D were either 0.02 ppm that constituted the selenium-deficient diet, 0.1 ppm that corresponded to approximately the normal level of this element in the human diet, 0.4 ppm that corresponded to approximately the level normally supplemented in human clinical trials and 2.25 ppm that represented a highly enriched selenium level. Subgroup E within each group contained 30 ppm selenium as TPSC that provided a very high level of selenium which was not further metabolized and could not be used in selenoprotein synthesis. Thus, this condition represented high levels of a selenocompound coupled with selenoprotein deficiency. The four major Groups contained mice with the following genotypes: 1) *TGFα*/*Trsp^tG37^*; 2) *Trsp^tG37^*/+; 3) *TGFα*/+; and 4) +/+ (see Table 1). The entire study extended over 18 months. The number of mice in each dietary group, average days they were on the diet, and their genotypes are summarized in Table 1.

The low-selenium Torula yeast-based diet [22] was supplemented with either 0, 0.1. 0.4 or 2.25 ppm of selenium as sodium selenite or 30 ppm selenium as TPSC by the vendor. The amount of selenium was measured in the non-supplemented diet and found to be 0.02 ppm. After approximately 120 days, *Trsp^tG37^*/+ and *TGFα*/*Trsp^tG37^* mice fed either the 0 selenium or 30 ppm selenium as TPSC supplemented diets began to display weakness in the hind legs, frequent loss of balance and a waltzing behavior that progressed to paralysis. Difficulties to right themselves after having been flipped on their backs indicated a more severe neurological defect; such mice were ultimately euthanized. A video showing the physical abilities of these animals was prepared (see Video S1).

To examine these groups more extensively, additional *Trsp^tG37^*/+, *TGFα*/*Trsp^tG37^* and +/+ mice were assigned each of the diet groups (see Table 2). These mice were maintained on the diets for 120 days before being euthanized for analysis.

**Table 2:** Multi-organ pyogranulomatous/granulomatous inflammation^a^.

	Genotype and Diet^b^						
	*TGFα*/*Trsp^tG37^*		*Trsp^tG37^*/*+*		*+*/*+*		*FVB*
Tissue	0.02 ppm Se (1A)	30 ppm Se asTPSC (1E)	0.02 ppmSe (2A)	30 ppm Se asTPSC (2E)	0.02 ppmSe (4A)	30 ppm Se asTPSC (4E)	Standard chow
Liver	1/18 (5.6)	3/17 (17.6)	13/19 (68.4)	11/17 (64.7)	1/5 (20)	1/5 (20)	0/3
Heart	1/12 (8.3)	3/9 (33.3)	3/13 (23)	2/9 (22.2)	0/3	0/3	0/3
Lung	0/13	1/11 (9.1)	7/18 (38.9)	3/12 (25)	0/3	1/3 (33.3)	0/3
Spleen	0/17	0/16	7/19 (36.8)	10/17 (58.8)	0/5	0/5	0/3
Small Intestine	8/13 (61.5)	7/16 (43.8)	13/18 (72.2)	9/17 (52.9)	2/5 (40)	1/5 (20)	0/3
Colon	2/14 (14.3)	0/14	8/14 (44.4)	4/17 (23.5)	0/5	0/5	0/3
Lymph node(mesenteric)	2/14 (14.3)	1/15 (6.7)	6/16 (37.5)	3/13 (23)	1/5 (20)	1/5 (20)	0/3

a Incidence of pyogranulomatous inflammation in mice maintained on the Torula yeast based diet. Animals were sacrificed at 120 days and various tissues examined for the occurrence of pyogranulomatous/granulomatous inflammation. Pyogranuloma incidence of groups for each organ were compared using Fisher’s Exact test (results given in text).

b Values shown represent incidence of pyogranulomatous inflammation/number of mice used in the study and the value in parenthesis is the percent incidence.

### Labeling of Selenoproteins, Isolation and Fractionation of tRNA and Selenium Analysis

Mice were injected intraperitoneally with 50 µCi of ^75^Se/g and euthanized 48 hrs after injection as described previously [13]–[15]. Liver was excised immediately, frozen in liquid nitrogen and stored at –80°C until ready for use. Liver tissue was homogenized, prepared for electrophoresis, electrophoresed, and the developed gel stained with Coomassie blue, dried and exposed to a PhosphorImager as described [13]–[15].

Total tRNA was isolated from pooled livers (n = 3) obtained from each subgroup of mice, aminoacylated with ^3^H-serine and 19 unlabeled amino acids in the presence of rabbit reticulocyte synthetases [23], and the resulting aminoacylated tRNA fractionated on a RPC-5 column [24] in the absence and then in the presence of Mg^++^ as given [13]–[15]. The techniques for assessing the amounts of Sec tRNA^[Ser]Sec^ expressed in liver from animals on the various diets relative to the total Ser tRNA population and the distributions of the two Sec isoforms, designated mcm^5^U and mcm^5^Um, have been detailed elsewhere [13]–[15].

The amount of selenium in plasma and in extracts of liver was determined by electrothermal-atomic absorption spectrometry as described [25].

### Pathology

Mice were euthanized with CO_2_ when they started to lose balance or failed to right themselves. As a high, early morbidity and mortality occurred in Group 1A, 1E, 2A and 2E on the selenium deficient and TPSC diets, complete necropsy examinations were performed with collection of all major organs and tissues as well as gross lesions. All remaining mice were sacrificed at 18 months. Tissues were fixed in 10% buffered neutral formalin. Blood was collected for selenium analysis immediately prior to euthanasia. Portions of gross nodular lesions that were suggestive of pyogranulomas were collected from a subset of mice with sterile technique for microbiology examination.

The following organs and tissues were processed, embedded in paraffin and stained with hematoxylin and eosin (H&E): heart, kidney, brain (1/2 sagittal section), mesenteric lymph node, spleen, liver (all lobes), lung with bronchus, Swiss roll of small intestine, Swiss roll of colon, femur with marrow, cross sections of cervical, thoracic and lumbar vertebrae with spinal cord, quadriceps and gastrocnemius muscle from one hind leg, and lesions designated by the pathologist. Histopathology evaluation was performed on all tissues as described above as well as on a subset of granulomas/pyogranulomas stained with the following special stains in an attempt to identify adventitious organisms: periodic acid Schiff (PAS), Ziehl Nielsen, acid fast, Brown and Hopps tissue Gram stain, Steiner stain, Gomori methenamine silver (GMS) and toluidine blue.

Gross lesions were cultured for aerobic and microaerophilic organisms. Several clinically ill mice were submitted to the Animal Health Diagnostic Laboratory for a thorough health status workup including bacteriology, *Helicobacter sp.* PCR (cecum), parvovirus PCR (feces, liver, spleen, kidney and intestine) and comprehensive serology for viral agents, *Mycoplasma pulmonis*, CAR bacillus and *Encephalitozoon cuniculi*. Sentinel female DBA/2NCr mice in the study animal room were on the same caging, bedding and water system as experimental mice. These sentinels were fed Rat and Mouse 18% Animal Diet (PMI Nutrition International, Brentwood, MO). During the course of this study, sentinels were routinely screened by the Animal Health Diagnostic Laboratory using microbiology, serology and histopathology to monitoring colony health. Organs examined histologically included cecum and colon (gut roll), small intestine (gut roll), spleen, lungs, heart and brain. Aliquots of each Harlan Teklad diet were cultured for bacterial and mycotic pathogens.

### Statistical Analyses

Data were statistically analyzed in SAS version 9.2 (SAS Institute Inc., Cary, NC). Incidence and multiplicity of liver tumors (adenoma, carcinoma, and adenoma plus carcinoma) and incidence of pyrogranulomas for each organ were compared between groups using Fisher’s exact test in PROC FREQ. Hepatic tumor numbers were compared between groups as a negative binomial variable using the logistic link function in PROC GLIMMIX. Fixed effects in the statistical model were genotype (Groups 1, 2, 3 and 4; Groups 2 and 4 were not included in the analysis of carcinoma number because the number of carcinomas were too low for obtaining an estimate), dietary selenium (Subgroups B, C and D; Subgroups A and E were not included in the analysis because Groups 1A, 1E, 2A, and 2E died prematurely resulting in an incomplete factorial design), and the interaction between genotype and dietary selenium. Orthogonal contrasts were constructed to evaluate the effects of *TGFα* genotype (Groups 1 and 3 versus Groups 2 and 4), of *Trsp^tG37^* genotype (Groups 1 and 2 versus Groups 3 and 4), and of the interaction of the two transgenes (Groups 1 and 4 versus Groups 2 and 3). In addition, the effect of high dietary Se concentrations was evaluated (Subgroups C and D versus Subgroup B). The effect of Se deficiency (Subgroups A and E) was tested in mice of Group 3. The effect of Se deficiency was evaluated by constructing orthogonal concentrations between mice receiving inadequate Se (Subgroups A and E) and mice receiving adequate Se (Subgroup B) or mice receiving supranutritional Se concentrations (Subgroups C and D). Data shown in tables are raw means and their standard deviation. It should also be noted that the limited number of tumor-bearing mice that did not carry the *TGFa* transgene and the premature death of most selenium-deficient mice, including four complete treatment groups, limited the statistical analysis of the liver tumor data.

Hepatic tumor sizes (total tumor area and average tumor size) of tumor bearing mice were analyzed using least square means procedures in PROC GLM. The sizes of two-dimensional tumors were calculated using the formula for a circle, and the sizes of three-dimensional tumors were calculated using the formula for a cylinder. The size of approximately 15% of the tumors, including all tumors from Group 4, could not be measured because tumors were measured after all other analyses were completed. Tumor area and size were normalized before statistical analysis using the natural logarithm transformation. Fixed effects in the statistical model were genotype (Groups 1 and 3; Groups 2 and 4 were not included because of the low tumor incidence in Groups 2 and 4), dietary selenium (Subgroups B, C and D; Subgroups A and E were not included in the analysis because Groups 1A and 1E died prematurely), and the interaction between genotype and dietary selenium. Data for tumor sizes are geometric means and their standard deviation. Statistical significance was declared at *P*≤0.05 and a tendency at 0.05<*P*≤0.10.

## Results

### Experimental Design

Considering significant variability in the outcomes of dietary intervention studies involving selenium in both the human population and animal models, we designed an in-depth controlled study to examine, separately and in combination, the effects of dietary selenium and selenoprotein status in cancer development. As a cancer driver, we used *TGFα*, a transgene that leads to increased hepatocarcinogenesis in the later stages of life [16]. The use of this “mild” cancer driver gene, combined with utilization of five different selenium diets allowed us to examine the impact of both metabolized and non-metabolized selenium compounds on hepatocarcinogenesis and make it as close to the human situation (wherein cancers develop slowly and their incidence increases with advanced age) as possible. In addition, to separate the effects of dietary selenium and selenoprotein status, we used transgenic mice carrying *Trsp^tG37^*. Such a design resulted in a large number of animal groups, which are summarized in Table 1.

*TGFα/Trsp^tG37^* and *Trsp^tG37^*/+ mice that were fed selenium-deficient or TPSC diets (Table 1, Subgroups 1A and 2A and 1E and 2E, respectively) suffered early deaths after being on these diets for only several months. Subgroups B–D that were maintained on diets with supplemented selenium and all mice in the Groups 3 and 4 showed low mortality and could be analyzed for hepatocarcinogenesis.

### Liver Tumorigenesis

Hepatocellular adenomas and carcinomas were the predominant form of tumors found, while only two hepatoblastomas and one hemangiosarcomas were detected in one mouse in Groups 1B and 3E, respectively. The data revealed four findings: 1) The *TGFα* transgene is a major driver of liver tumorigenesis, while the liver tumor incidence in mice not carrying the *TGFα* transgene after 18 months is low (Table 3). Mice harboring the *TGFα* transgene (Groups 1 and 3) had a significantly greater formation of adenomas, carcinomas, and both adenomas and carcinomas than mice lacking the *TGFα* transgene (Groups 2 and 4; all p<0.01; Table 3). The effect of the *TGFα* transgene on liver tumorigenesis was not significantly modified by the presence or absence of the *Trsp^tG37^* transgene.

**Table 3:** Incidence and multiplicity of liver tumors^a^.

				Tumor type - Incidence^b^			Multiplicity^c^	
Genotype	Group	# Mice in which livers were examined	# Mice with tumors	Hepatocellularadenoma	Hepatocellular carcinoma	Adenoma and carcinoma	Total hepatocellular adenomas	Total hepatocellular carcinomas
*TGFα*/*Trsp^tG37^*	1B	14	12	8/14 (57%)	7/14 (50%)	3/14 (21%)	10	16
	1C	26	17	11/26 (42%)	12/26 (46%)	6/26 (23%)	16	23
	1D	19	14	9/19 (47%)	10/19 (53%)	5/19 (26%)	12	18
*Trsp^tG37^*/*+*	2B	13	4	4/13 (31%)	0/13 (0%)	0/13 (0%)	4	0
	2C	19	3	3/19 (16%)	0/19 (0%)	0/19 (0%)	3	0
	2D	18	7	6/18 (33%)	1/18 (6%)	0/18 (0%)	10	1
*TGFα*/*+*	3A	21	12	7/21 (33%)	8/21 (38%)	3/21 (14%)	7	10
	3B	21	17	12/21 (57%)	11/21 (52%)	6/21 (29%)	15	14
	3C	20	15	12/20 (60%)	8/20 (40%)	5/20 (25%)	16	11
	3D	19	14	9/19 (47%)	12/19 (63%)	7/19 (37%)	14	16
	3E	18	12	8/18 (44%)	10/18 (56%)	6/18 (33%)	10	13
*+*/*+*	4A	10	0	0/10 (0%)	0/10 (0%)	0/10 (0%)	0	0
	4B	6	2	2/6 (33%)	1/6 (17%)	1/6 (17%)	3	2
	4C	8	1	1/8 (13%)	0/8 (0%)	0/8 (0%)	2	0
	4D	9	1	1/9 (11%)	1/9 (11%)	1/9 (11%)	1	1
	4E	8	1	0/8 (0%)	1/8 (13%)	0/8 (0%)	0	1

a Tumor incidence and multiplicity of groups were compared using Fisher’s Exact test (see text).

b Fraction of mice with each cancer type. Percent of mice with the tumor type shown in parentheses. In addition to adenomas and carcinomas, one mouse in Group 1B had two hepatoblastoma and one mouse in Group 3E had one hemangiosarcoma.

c Total number of hepatocellular adenomas or carcinomas in all mice.

2) The *Trsp^tG37^* transgene may promote formation of adenomas but not carcinomas, since mice with the *Trsp^tG37^* gene alone (Group 2) had more adenomas than carcinomas, while the other three genotypes had similar numbers of adenomas and carcinomas (p<0.03; Table 3).

3) In mice carrying the *TGFα* transgene, high dietary selenium levels appear to inhibit liver tumor growth (Table 4). Compared to tumor-bearing mice fed 0.1 ppm selenium, tumor-bearing mice fed 0.4 or 2.25 pm selenium had smaller adenomas (p = 0.03 for both groups combined and for 0.4 ppm only) and carcinomas (p = 0.02 for both groups combined and p = 0.03 for 0.4 ppm only) and had a smaller total area of adenomas (p = 0.10 for both groups combined and p = 0.06 for 0.4 ppm only) and carcinomas (p = 0.04 for both groups combined and p = 0.05 for 0.4 ppm only). No significant differences in adenoma and carcinoma incidence, multiplicity, and number were observed between dietary groups.

**Table 4:** Area and size of liver tumors^a^.

		Tumor Number (tumor/mouse)		Total Tumor Area (in mm^3^/per tumor bearing mouse)^b^		Tumor Size (in mm^3^/tumor per tumor bearing mouse)^b^	
Genotype	Group	Adenomas	Carcinomas	Adenomas	Carcinomas	Adenomas	Carcinomas
*TGFα*/*Trsp^tG37^*	1B	0.7±0.8	1.1±1.4	40±14^b^	556±121	36±12	331±55
	1C	0.6±0.9	0.9±1.2	10±6	272±69	8±5	174±40
	1D	0.6±0.8	0.9±1.0	15±9	388±33	12±8	229±20
*Trsp^tG37^*/*+*	2B	0.3±0.5	0	10±11	0	10±11	0
	2C	0.3±0.4	0	10±2	0	10±2	0
	2D	0.6±0.9	0.06±0.24	15±4	79	13±3	79
*TGFα*/*+*	3A	0.3±0.5	0.5±0.7	5±4	222±35	5±4	187±35
	3B	0.7±0.7	0.7±0.7	23±8	469±106	20±8	388±106
	3C	0.8±0.8	0.6±0.8	18±9	188±20	15±7	145±20
	3D	0.7±1.0	0.8±0.8	21±9	191±50	14±4	155±50
	3E	0.6±0.8	0.7±0.8	30±10	170±38	27±9	142±38
*+*/*+*	4A	0	0	0	0	0	0
	4B	0.5±0.8	0.3±0.8	NA^c^	NA^c^	NA^c^	NA^c^
	4C	0.3±0.7	0	NA^c^	0	NA^c^	0
	4D	0.1±0.3	0.1±0.3	NA^c^	NA^c^	NA^c^	NA^c^
	4E	0	0.3	0	NA^c^	0	NA^c^

a Tumor number (see Table 3), size, and area were assessed at necropsy and compared using least square means procedures (see text). Tumor numbers are shown as raw means ± standard deviations. The sizes of approximately 15% of tumors were not measured, including all tumors for Group 4.

b Tumor area and size are shown as geometric means ± standard deviations.

c NA, no size measure for tumors available.

4) In mice carrying the *TGFα* transgene, selenium deficiency may inhibit liver tumor size (Table 4). Compared to carcinoma-bearing mice fed 0.1 ppm selenium, carcinoma bearing mice fed no supplemental selenium or TPSC had smaller carcinomas and a smaller total area (both p = 0.05). No significant differences in adenoma and carcinoma incidence, multiplicity, and number or adenoma area and size were observed in response to selenium deficiency.

### Pathology of Trsp^tG37^/+ mice Maintained on Selenium Diets

Histopathology analysis of *TGFα*/*Trsp^tG37^* and *Trsp^tG37^* mice maintained on the selenium-deficient or TPSC diets showed a high incidence of pyogranulomas in several tissues. Pyogranulomatous and/or granulomatous lesions occurred in multiple tissues, including those from control (FVB/CD1) mice. *Trsp^tG37^* mice fed selenium-deficient or TPSC diets (Groups 2A, 2E) had the most widely disseminated distribution and highest incidences of these lesions (Table 2).

**Figure 1:**
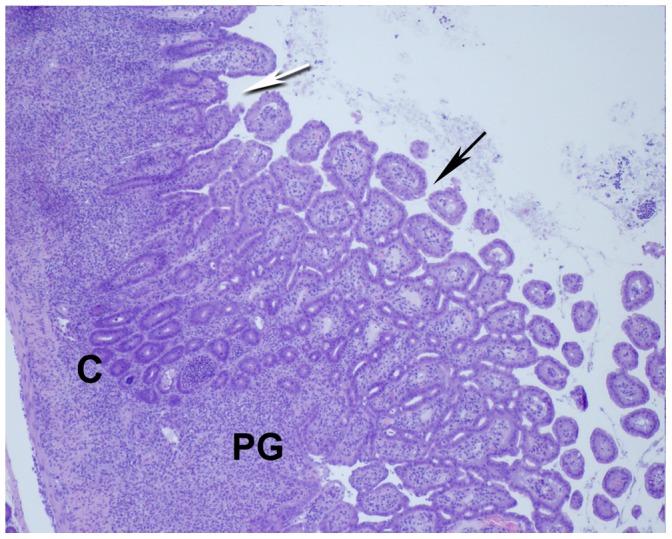
Pyogranulomatous inflammation of the small intestine. Cross section showing diffuse pyogranulomatous inflammation involving the mucosa and muscularis: Areas of highly populated pyogranulomous inflammation (PG), overlying villi of the mucosa are shortened (white arrow) and fused (black arrow) and the crypts (C) are hyperplastic (shown at 4X magnification). Details of processing and staining of the tissue are provided in Materials and Methods.

The highest incidence of pyogranulomatous lesions occurred in the small intestine. They were present throughout the tissue and were most extensive in the ileum (Fig. 1). Pyogranulomas were located in the deep mucosa, submucosa, muscularis and/or serosa of this tissue. Crypts overlying the pyogranulomas were hyperplastic and villi were fused or atropic (Fig. 1) and ulceration was uncommon.

**Figure 2:**
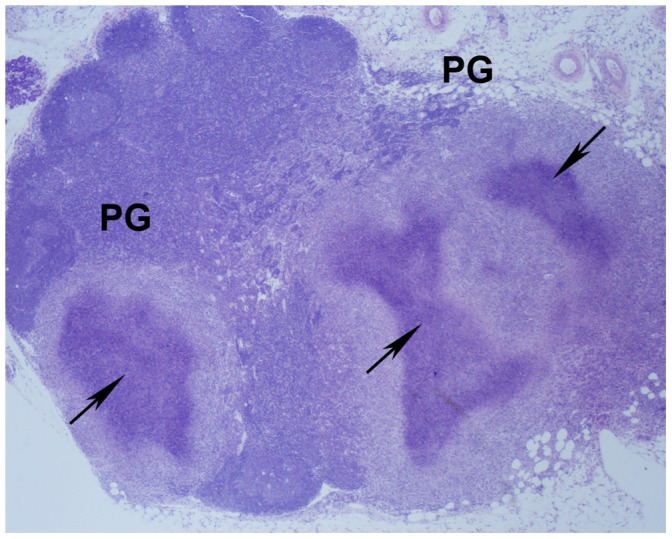
Pyogranulomatous inflammation of the mesenteric lymph node. Cross section showing confluent pyogranulomatous inflammation (PG) with caseous necrotic centers (arrows) (shown at at 4X magnification). Details of processing and staining of the tissue are provided in Materials and Methods.

**Figure 3:**
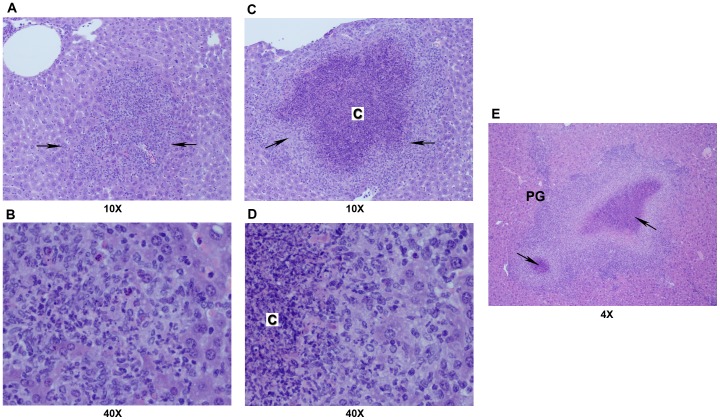
Pyogranulomatous inflammation of liver. Sections with varying degrees of severity of pyogranulomatous lesions: in (A) and (B), focal pyogranuloma (arrows) without caseous necrosis (shown at 10 and 40X magnification, respectively); in (C) and (D), a larger pyogranuloma (arrows) with a caseous necrotic center (designated as C; shown at 10 and 40X magnification, respectively); and in E, confluent pyogranulomas with caseous necrotic centers (arrows; shown at 4X magnification). Details of processing and staining of the tissue are described in Materials and Methods.

**Figure 4:**
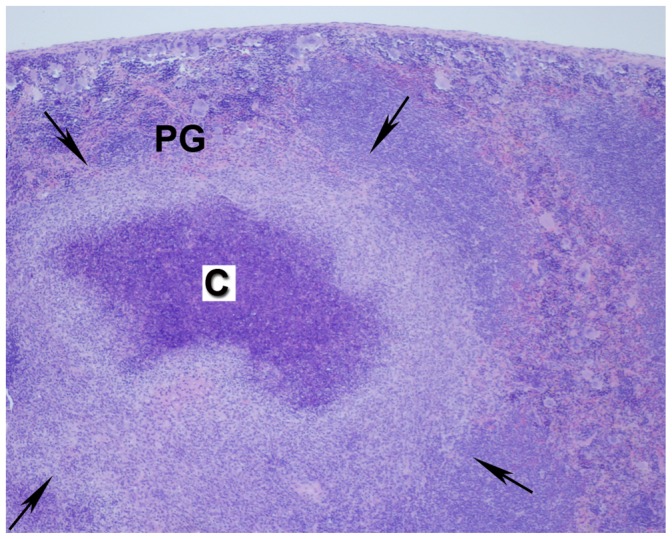
Pyogranulomatous inflammation of the spleen. Cross section showing pyogranulomatous inflammation (PG,) wherein arrows delineate borders of the pyogranuloma. The center has caseous necrosis (designated by C; shown at 4X magnification). Details of processing and staining of the tissue are described in Materials and Methods.

Pyogranulomas in the large bowel, especially the colon, occurred in Groups 1A, 2A and 2E. Mesenteric lymph nodes had pyogranulomatous inflammation in all groups of mice with highest incidences in *Trsp^tG37^* mice (Groups 2A, 2E) (Fig. 2). Liver had single to multiple foci of inflammation that ranged from microscopic to gross nodules. Pyogranulomas had central foci of neutrophils as shown in Figures 3A and 3B or a caseous necrotic core (Fig. 3C, 3D). Liver lesions were more severe in Groups 1 and 2E because they were more extensive and had necrotic centers (Fig. 3E). Pyogranulomatous lesions in the spleen occurred only in *Trsp^tG37^* mice group. Splenic pyogranulomas were often detected grossly and were discrete or confluent (Fig. 4). They were composed of a core of caseous necrosis that was surrounded by an admixture of macrophages, neutrophils and other mononuclear cells (Fig. 4).

As shown in Table 2, *Trsp^tG37^* mice fed a selenium-deficient diet (Group 2A) had a statistically greater incidence of pyogranulomatous inflammation in liver, spleen, lung and colon than *TGFα*/*Trsp^tG37^* on the same diet (Group 1A) (p<0.05). Pyogranulomatous inflammation in liver and spleen of *Trsp^tG37^* mice fed a 30 ppm selenium as TPSC diet (Group 2E) was also greater than *TGFα*/*Trsp^tG37^* mice on the same diet (p<0.05) (Group 1E). Such pyogranulomatous formation in Groups 1 and 2 did not differ from the corresponding control mice (Group 4). The exception was spleen in *Trsp^tG37^* mice fed the 30 ppm selenium as TPSC diet (Group 2E) that had a significantly greater incidence of pyogranulomatous formation (p<0.05) than control mice fed the same diet (Group 4E).

### Pyogranuloma Occurrence is not Associated with Common Infectious Agents

Since granulomatous inflammation in humans, including those with chronic granulomatous disease [26], and in other animals, are due to inter-current infection (see reviews [27]–[29]), we examined pyogranulomas in intestine, spleen and liver for such an agent. Numerous techniques as discussed in Materials and Methods were used. A spectrum of special stains, transmission electron microscopy and microbiology failed to reveal viral, bacterial, mycotic or protozoan organisms. The experimental diet was based on Torula yeast and glucose monohydrate, and PAS-positive yeast forms could be detected sporadically in the lumen of the small intestine. However, the small bowel and samples of the diet were negative for yeast in culture. Bacterial pathogens were not detected in cultures of the diet. Finally, sentinel mice in the study room had no bacterial or viral pathogens and no histologic evidence of pyogranulomas. We conclude from these analyses that the pyogranulomas did not have detectable infectious etiology, i.e., they were sterile. The lesions may nevertheless have impaired organ function that may contribute to morbidity (see discussion).

### Selenium Levels

To verify the effects of dietary selenium and transgene expression, selenium levels were measured in liver and plasma of *TGFα*/*Trsp^tG37^* and *Trsp^tG37^* mice, Subgroups A, B and E, respectively, and of control, +/+ mice, Subgroup B (Table 5). Liver and plasma were assayed for three animals from each group. Selenium levels were reduced dramatically in Groups 1 and 2 that contained *Trsp^tG37^* mice on selenium-deficient and TPSC diets that contributed to their poor health, neurological impairment, pyogranuloma formation and early death. Selenium levels in liver of control mice fed selenium deficient or TPSC diets were also low but not as low as those mice on the corresponding diets carrying the *Trsp^tG37^* transgene.

**Table 5:** Selenium levels in liver and plasma of *TGFα/Trsp^tG37^*, *Trsp^tG37^/+* and *+/+* mice^a^.

			Selenium (ppb)	
Genotype	Group	Diet	Liver (wet wt)	Plasma
*TGFα*/*Trsp^tG37^*	(1A)	0.02 Se	59.9+/−9.0	17.7+/−8.6
*TGFα*/*Trsp^tG37^*	(1B)	0.1 Se	198.1+/−22.6	131.9+/−8.1
*TGFα*/*Trsp^tG37^*	(1E)	30 TPSC	125.1+/−0.8	18.7+/−10.5
*Trsp^tG37^*/*+*	(2A)	0.02 Se	87.1+/−12.5	ND^b^
*Trsp^tG37^*/*+*	(2B)	0.1 Se	271.1+/−8.8	127.1+/−6.0
*Trsp^tG37^*/*+*	(2E)	30 TPSC	82.7+/−5.3	3.9+/−1.7
*+*/*+*	(4A)	0.02 Se	142.2+/−36.9	NM^c^
*+*/*+*	(4B)	0.1 Se	1206.3+/−233.6	260+/−48.5
*+*/*+*	(4E)	30 TPSC	281.9+/−23.9	NM^c^

a Selenium levels were measured in triplicate as given in Materials and Methods and values represent the mean and standard error of three different mice in each genotype.

b ND = none detected.

c NM = not measured.

### Metabolic ^75^Se-labeling and Sec tRNA^[Ser]Sec^ Levels

**Figure 5:**
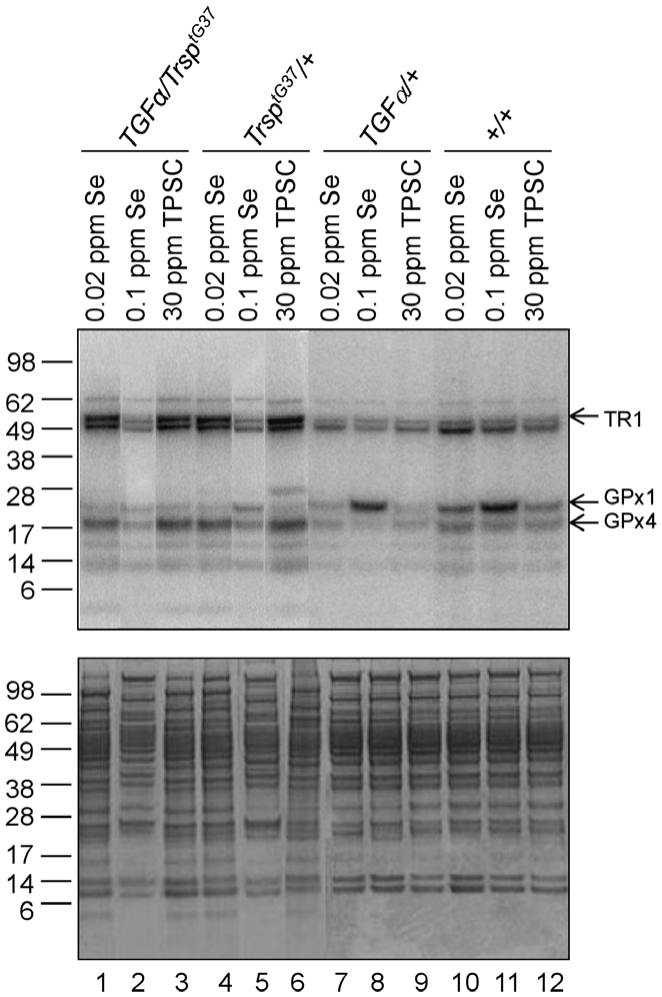
Labeling of selenoproteins in liver. Mice were labeled with ^75^Se, protein extracts prepared, aliquots of the extracts electrophoresed on denaturing gels, the developed gels stained with Coomassie Blue to use as a loading control (lower panel) and ^75^Se-labeled selenoproteins detected using a PhosphorImager (upper panel). Molecular weights of proteins are indicated on the left of the panels and selenoprotein identity by arrows on the right of the upper panel. Details of the procedures are described in Materials and Methods.

To examine additional indicators of selenium and selenoprotein status, we metabolically labeled selenoproteins with ^75^Se in a subset of mice (Fig. 5) and also quantified Sec tRNA^[Ser]Sec^ levels in livers (Table 6). Mice that were maintained on 0.02 ppm selenium, 0.1 ppm selenium or 30 ppm TPSC diets and encoded the *Trsp^tG37^* transgene, with or without the *TGFα* transgene, were labeled with ^75^Se at 120 days. Control mice included the +/+ animals and those encoding *TGFα* (Fig. 5). The pattern of ^75^Se-labeling in liver from mice encoding *Trsp^tG37^* (lanes 1, 3, 4 and 6) was similar with the exception of mice maintained on the 0.1 ppm selenium diet (lanes 2 and 5). The labeling of selenoproteins was diminished in animals maintained on the 0.1 ppm selenium diet compared to the 0.02 ppm selenium and TPSC diets which was due to the fact that the ^75^Se was diluted by the 0.1 ppm selenium. It was not surprising that TR1 and GPx4 were expressed in approximately equal amounts in liver of mice maintained on Se deficient or TPSC diets, since these selenoproteins are synthesized by the mcm^5^U isoform which is less sensitive to selenium status than the mcm^5^Um isoform [5]. The higher levels of expression of TR1 and GPx4 in mice carrying *Trsp^tG37^*compared to mice lacking this transgene (compare lanes 1–6 to lanes 7–12) is undoubtedly due to the fact that stress-related selenoproteins are poorly expressed in mice synthesizing mutant tRNA^[Ser]Sec^ resulting in higher levels of housekeeping selenoprotein expression [5], [13]–[15].

**Table 6:** Levels and distributions of the Sec tRNA^[Ser]Sec^ isoforms in liver of mice^a^.

				Distribution		
Genotype	Diet (ppm Se)	% i6A^−^	% (total)	mcm^5^U	mcm^5^Um	mcm^5^Um/mcm^5^U
*TGFα/Trsp^tG37^*	0.02	15.89%	1.26	74.72	25.28	0.34
	0.1	17.93%	1.62	67.04	32.96	0.49
	30 (TPSC)	14.49%	1.63	83.77	16.23	0.19
*Trsp^tG37^/+*	0.02	14.92%	1.24	82.90	17.10	0.21
	0.1	13.04%	1.24	74.80	25.20	0.34
	30 (TPSC)	14.90%	1.59	82.11	17.89	0.22
*TGFα/+*	0.02	0%	5.15	62.91	37.09	0.59
	0.1	0%	5.44	44.52	55.48	1.25
	30 (TPSC)	0%	3.90	64.61	35.39	0.55
*+/+*	0.02	0%	3.85	62.41	37.59	0.60
	0.1	0%	2.32	55.91	44.09	0.78
	30 (TPSC)	0%	3.23	62.51	37.49	0.60

a Bulk tRNA was isolated from liver of the four mouse lines (column 1) that were maintained on different diets (column 2), the tRNA aminoacylated with ^3^H-serine, the resulting labeled seryl-tRNA fractionated on a RPC-5 column, the percent of the i^6^A^−^and wild type seryl-tRNA^[Ser]Sec^ isoforms within the total seryl-tRNA^[Ser]Sec^ population determined (columns 3 and 4, respectively), the distributions of mcm^5^U and mcm^5^Um assessed as the percent of wild type mcm^5^U and mcm^5^Um seryl-tRNA^[Ser]Sec^ isoforms within the total seryl-tRNA population (columns 5 and 6, respectively) and ratio of mcm^5^Um to mcm^5^U determined as described in Materials and Methods.

GPx1, on the other hand, is poorly expressed in mice synthesizing mutant tRNA^[Ser]Sec^ as these mice are selenium deficient and express the mcm^5^Um isoform in low levels [5], [13]–[15]. As noted in the Introduction, stress-related selenoproteins require the mcm^5^Um isoform for their expression. Furthermore, the level of GPx1 in liver from *TGFα* and control animals on the 0.1 ppm selenium diet appeared enhanced relative to other selenoproteins and compared to GPx1 in mice on the selenium-deficient and TPSC diets (compare lanes 8 and 11 to lanes 7, 9–10 and 12). *GPx1* mRNA is labile compared to many other selenoprotein mRNAs under selenium-deficient conditions [30] which also accounts for its obviously greater expression in mice on the 0.1 ppm selenium diets relative to other selenoproteins in liver as well as in animals on the selenium-deficient and TPSC diets.

Sec tRNA was examined in liver from mice that had been maintained on the 0.02 ppm selenium, 0.1 ppm selenium or TPSC diets (Table 6). The levels of the mutant isoform generated from 20 copies of *Trsp^tG37^* ranged from ∼15% to ∼18% of the total seryl-tRNA population. As shown in an earlier study, the synthesis of the A37G isoform resulted in decreased expression of the normal Sec tRNA^[Ser]Sec^ population compared to mice not carrying *Trsp^tG37^* [13]. The distributions of the mcm^5^U and mcm^5^Um isoforms were also influenced by the presence of selenium [13]–[15] or TPSC in the diet. The two Sec tRNA^[Ser]Sec^ isoforms shifted towards more mcm^5^U in most cases in mice maintained on selenium-deficient diets. This observation was most apparent in *TGFα* and control mice (i.e., those mice not encoding *Trsp^tG37^*). The results shown in Table 6 were consistent with data obtained in numerous earlier studies examining the impact of selenium deficiency and/or the A37G mutant tRNA isoform on the levels and distributions of the two Sec tRNA^[Ser]Sec^ isoforms in mammalian tissues [13]–[15] or in mammalian cells grown in culture [31].

## Discussion

The present study was undertaken to elucidate the role of dietary selenium and selenoproteins in hepatocarcinogenesis in mice carrying *TGFα*, which is a liver cancer driver transgene [16], and/or a mutant *Trsp^tG37^* transgene, that disrupts stress-related selenoprotein biosynthesis [13]–[15] and enhances carcinogenesis in other organs [10], [11]. Mice encoding either *TGFα*/*+*, *Trsp^tG37^*/*+* or both transgenes, and control mice that were all in the same genetic background were placed on identical diets except that the diets contained either 0.02, 0.1, 0.4 or 2.25 ppm selenium as sodium selenite or 30 ppm selenium as TPSC. Since mice carrying *Trsp^tG37^* were already selenium-deficient due to the loss of stress-related selenoprotein expression [13], these mice became extremely selenium-deficient on diets lacking selenium supplementation or containing TPSC and developed an early and unexpected morbidity and mortality.

Tumor development was assessed in livers of mice that survived longer than mice carrying *Trsp^tG37^* that were maintained on the selenium deficient or TPSC diets. The greater incidence of hepatocellular tumors occurred in mice carrying *TGFα*. The co-presence of *Trsp^tG37^*, which is known to generate selenium deficiency and alter stress-related selenoprotein expression [13]–[15], had no statistically significant influence on enhancing tumor formation. Thus, selenium deficiency induced by the *Trsp^tG37^* Sec tRNA^[Ser]Sec^, or through diet, did not appear to influence liver tumor incidence or number in mice encoding *TGFα* (Table 3). However, *TGFα* encoding mice fed recommended levels of selenium (0.1 ppm) had larger adenomas and carcinomas than mice fed supranutritional levels of selenium (0.4 or 2.25 ppm) and these *TGFα* mice had larger carcinomas than selenium-deficient mice (Table 4). In comparison, *TGFα/c-Myc* bi-transgenic mice maintained on either selenium-deficient or highly enriched selenium (2.25 ppm) diets were found to have a lower incidence and number of liver tumors than the corresponding mice maintained on 0.1 or 0.4 ppm selenium [8]. Tumor size and total area was not reported in the latter study. These latter bi-transgenic mice developed tumors within 6 months and *c-Myc*, which is also known to be a liver cancer driver [32], along with *TGFα*, shortened the time of tumor development considerably compared to *TGFα* alone. The advantage of using *TGFα* as the sole cancer driver gene compared to the bi-transgenic cancer driver model is the additional time allotted by the single transgenic model to develop tumors that in turn permitted the control (non-bearing *TGFα*) mice to also develop tumors. As discussed below (also see Table 3), these “controls” (control and *Trsp^tG37/+^* mice) developed tumors which were mostly adenomas, particularly in *Trsp^tG37/+^* mice, demonstrating that the “controls” may also get tumors, but at a far lower rate. Furthermore, adenomas are much less detrimental than carcinomas.

The data in the *TGFα/c-Myc* bi-transgenic mice study [8] suggested that alterations in selenoprotein expression may suppress or enhance tumorigenesis depending on cell type or genotype. Clearly, the influence of selenium metabolism, including selenoprotein expression, on tumor incidence appears to be different in liver ([8], [33] and data herein) compared to other organs such as colon [10], prostate [11] and breast [12], wherein the loss of stress-related selenoprotein or total selenoprotein expression results in a dramatic increase in tumor expression. Furthermore, the loss of TR1 expression in mouse liver results in a dramatic increase in tumor formation compared to TR1 expressing mice [33], whereas mice carrying a knockout of Sep15 have a lower incidence of carcinogen-induced, pre-neoplastic colon malignancy [34]. TR1, therefore, does not appear to have a role in promoting liver neoplasia (see [35] for review on TR1’s role in promoting certain cancers), but apparently does have a role in protecting this organ from hepatocarcinogenesis [33].

Both the control and *Trsp^tG37/+^* mice appeared to develop more adenomas than *TGFα/+* or bi-transgenic mice. Overall, *TGFα* played a far more dominant role in liver tumor induction than selenium deficiency or reduced stress-related selenoprotein expression in the present study.

Histopathologic analyses of tissues from mice encoding *Trsp^tG37^* and maintained on diets lacking selenium supplementation or containing TPSC revealed a high incidence of pyogranulomatous inflammation. Pyogranulomatous inflammation was found in numerous tissues and organs including liver, heart, lung, spleen, small and large intestine and mesenteric lymph nodes. Whether pyogranulomas play a role in the early death of transgenic mice remains unclear, but is certainly possible. Interestingly, the incidence of pyogranulomas was significantly greater in *Trsp^tG37^* mice maintained on the 0.02 ppm selenium or 30 ppm TSPC diet than in the bi-transgenic mice on the corresponding diets. Thus, it appears that *TGFα* has an overall ameliorating effect on pyogranuloma formation in liver and spleen, and possibly lung, colon and lymph nodes. The reason for the greater incidence of pyogranuloma formation in some tissues observed in *Trsp^tG37^* mice compared to bi-transgenic mice is not clear.

The occurrence of pyogranulomas in mice and other species is often closely associated with co-infecting agents such as helminths and their ova, fungi, protozoa and bacteria [27]–[29]. Chronic granulomatous disease in humans is a group of disorders characterized by a defect in phagocytes (neutrophils, macrophages, other mononuclear cells) resulting in decreased superoxide formation and inability to contain infectious pathogens and opportunistic agents. Granulomatous inflammation then occurs in many organs. In a number of these patients, infections agents could not be isolated from granulomas [26]. An in-depth analysis of the pyogranulomas in study mice failed to reveal any co-infecting agents. Therefore, it is tempting to propose that the occurrence of the large number of pyogranulomas observed in affected mice herein was due to selenium deprivation compounded with at least a partial loss of selenoprotein expression, affecting phagocytosis and innate immunity [36], [37].

The neurological phenotype of the selenium-deficient mice is reminiscent of the neurological impairment experienced by selenoprotein P knockout mice and these animals died if they were not given selenium supplementation in their diets [18], [20]. Brain pathology in our mice revealed no specific findings, but silver staining was not performed. Therefore, axonal pathology as in selenoprotein P-deficient (*Sepp^−/−^*) mice cannot be ruled out [20]. Similarly, mice lacking neuronal selenoproteins because of neuron-specific inactivation of tRNA^[Ser]Sec^ expression suffered neurodegeneration [21]. A video showing the neurological phenotype encountered by the affected mice carrying *Trsp^tG37^* and maintained on the selenium-deficient diet is shown in Video S1. We have observed a similar phenotype in *Sepp^−/−^* mice maintained on a 0.15 ppm Se diet [18], [20]. These mice, after a fairly normal development, start to exhibit a movement disorder with a wide, dystonic gait. When fallen from a rotarod device, Sepp^−/−^ mice frequently showed the same failure to right themselves as shown in the accompanying video. The late occurrence of the phenotype in *Trsp^tG37^* mice suggests a degenerative rather than a developmental mechanism and is thus dissimilar to *Sepp^−/−^* mice. The exact pathology, however, remains unknown.

Selenium deficiency decreases the expression of the mcm^5^Um Sec tRNA^[Ser]Sec^ isoform in mammalian tissues and organs [13]–[15], in mammalian cells in culture [31], and in stress-related selenoproteins [13]–[15]. In addition, the presence of mutant Sec tRNA^[Ser]Sec^ has also been shown to induce selenium deficiency, alter the distribution of the Sec tRNA^[Ser]Sec^ population and affect selenoprotein expression. The pattern of ^75^Se-labeling of selenoproteins and the distribution of Sec tRNA^[Ser]Sec^ isoforms in the presence of a mutant Sec tRNA^[Ser]Sec^ and/or in the presence of very low or adequate levels of selenium were observed in the current study to be similar as those found in previous studies [13]–[15]. The relation between selenium deficiency and Sec tRNA^[Ser]Sec^ isoform distribution and selenoprotein expression has been reviewed in detail elsewhere [38].

In conclusion, the extensive hepatocarcinogenesis mouse study reported in this paper revealed the complexity of the role of selenium in cancer. Clearly, cancer type, nature of mutations that drive cancer, timing of cancer development, levels of selenium in the diet and selenoprotein status may all affect the outcome, which is not readily predictable. In this regard, it is particularly troubling that extensive human clinical trials have been carried out and are currently ongoing without a better understanding of the role of selenium and selenoprotein biology in health and development. If what is known from animal models is transferable to human cancer, then one may expect widely different effects of dietary selenium on the development of different subsets of cancers. Since we now know that specific selenoproteins may promote, as well as prevent cancer [35], [39], it would seem imperative that a better understanding of the split personalities of these selenoproteins, and of selenium metabolism as a whole, be obtained before further human clinical trials are undertaken.

## Supporting Information

Video S1 **Representative control (** ***+/+*** **) and i6A deficient (** ***Trsp^tG37^/+*** **) mice maintained on a 0.02 ppm Se diet were filmed to demonstrate the neurological phenotype observed in the** ***Trsp^tG37^/+*** **mice.**
(MPG)
